# Preparing your intensive care unit for the COVID-19 pandemic: practical considerations and strategies

**DOI:** 10.1186/s13054-020-02916-4

**Published:** 2020-05-11

**Authors:** Ken Junyang Goh, Jolin Wong, Jong-Chie Claudia Tien, Shin Yi Ng, Sewa Duu Wen, Ghee Chee Phua, Carrie Kah-Lai Leong

**Affiliations:** 1 Department of Respiratory and Critical Care Medicine, Singapore General Hospital, Outram Road, Singapore, 169608 Singapore; 2 Division of Anaesthesiology, Singapore General Hospital, Singapore, Singapore

**Keywords:** Coronavirus disease 2019, SARS-CoV-2, Critical care, Infection control, Pandemic preparedness

## Abstract

The coronavirus disease 2019 (COVID-19) has rapidly evolved into a worldwide pandemic. Preparing intensive care units (ICU) is an integral part of any pandemic response. In this review, we discuss the key principles and strategies for ICU preparedness. We also describe our initial outbreak measures and share some of the challenges faced. To achieve sustainable ICU services, we propose the need to 1) prepare and implement rapid identification and isolation protocols, and a surge in ICU bed capacity; (2) provide a sustainable workforce with a focus on infection control; (3) ensure adequate supplies to equip ICUs and protect healthcare workers; and (4) maintain quality clinical management, as well as effective communication.

## Background

Coronavirus disease 2019 (COVID-19) caused by severe acute respiratory syndrome coronavirus 2 (SARS-CoV-2) has rapidly developed into a worldwide pandemic [1, 2]. Initial studies from China reported a high incidence of acute respiratory distress syndrome (ARDS) (17–29%) and critical illness (23–32%) among hospitalised patients [3–6]. Similar rates of critical illness (16%) were also reported in Lombardy, Italy [7]. While these incidence rates are difficult to interpret and may be overestimated due to differences in availability of diagnostic testing, surveillance resources, and outpatient management of patients with mild illness, it appears that many patients who do develop critical illness do not survive. The reported 28-day ICU mortality rates are alarmingly high (62%) [4], and greater than mortality rates seen with severe ARDS [8]. While the estimated case fatality rate of COVID-19 (3–4%) [9] is lower compared to Middle East respiratory syndrome (MERS) (34%) and severe acute respiratory syndrome (SARS) (11%), deaths from COVID-19 has already far exceeded the combined deaths from MERS and SARS [10].

ICUs will be simultaneously challenged on multiple fronts. These include resource limitations, infection control, protection of healthcare workers (HCWs), and adaptation of services to a rapidly evolving pandemic situation. During the early phase of the outbreak in Wuhan, China, shortages in equipment meant that 75% of the deceased did not receive mechanical ventilation [11]. ICU resources in Lombardy, Italy, are also reported to be overwhelmed [7]. Clearly, the ability to maintain sustainable critical care services is a key consideration for all healthcare systems.

Singapore was among the first countries to experience an outbreak of COVID-19 [12], with approximately 10–15% of patients requiring invasive mechanical ventilation [13]. In this article, we aim to describe the critical care response in the largest academic tertiary medical centre in Singapore. The main targets to achieve are to (1) prepare and implement rapid identification and isolation protocols, and a surge in ICU bed capacity; (2) provide a sustainable workforce with a focus on infection control; (3) ensure adequate supplies to equip ICUs and protect HCWs; and (4) maintain quality clinical management, as well as effective communication. We review strategies employed with regard to ICU “SPACE”, “STAFF”, “SUPPLIES”, and “STANDARDS” (Table 1) [14, 15].

**Table 1:** Summary of considerations and strategies to maintain ICU capacity and services

	Containment or alert phase*	Pandemic or crisis phase*
Scenario	Limited community spread isolated to individuals or clusters	Sustained widespread community transmission
Key strategy	Containment and preparedness	Mitigation and containment
SPACE	Designate an isolation ICU, with negative pressure AIIR	
	Rapid identification and isolation of suspected/known COVID-19 cases	
	Ensure access to rapid diagnostic testing (e.g. laboratory facilities)	
	Initiate planning for surge ICU bed capacity	
		Utilise normal pressure ICU beds or existing monitored beds (e.g. OT, PACU, high dependency, endoscopy suites, emergency department)
		Alternative: cohort beds with physical barriers (e.g. curtains) in between patients
		Ensure timely step-down of stable patients with deisolation protocol
		Mass critical care: triaging protocol for patients with consideration for available resources, ethical principles, and public engagement
STAFF	Staff segregation into ‘frontline’ teams	
	Implement strict infection prevention and control measures	
	Education of HCWs on infection control measures with *just-in-time* N95 fit testing	
	In situ, *just-in-time* simulation training with before-and-after multidisciplinary peer-review processes	
	Periodic re-training of HCW on infection control measures	
	Staff surveillance (e.g. temperature monitoring) and access to designated staff clinics	
	Ensure dissemination of timely and factual information and establish two-way communication	
	Provide helplines and psychological support, temporary staff quarters, gratitude messages from hospitals and public	
	Initiate ICU hands-on training for non-critical care nurses and ICU refresher courses for HCW using online materials and instructional videos	
		Minimise unnecessary procedures and transport
		Increase manpower capacity by changing work structure (e.g. extra shifts or work hours) and restricting leave
		Suspend elective procedures and non-essential services
		Redeployment of HCW with critical care experience from other departments into ICUs
		Consider reducing nurse- and doctor-to-patient ratios
		Mass critical care: reassign non-intensive care HCW from other departments to support essential services, with ICU nurses providing a supervisory role
SUPPLIES	Ensure adequate supply and stockpiles of PPE, essential consumables, medication, and equipment	
	Source for alternative supply channels for supplies and equipment; consider extended use of supplies/consumables where safe to do so and rationalise use of essential medications	
	Switch to single-use items (e.g. disposable bronchoscopes)	
	Segregate equipment (e.g. designated ultrasound machines)	
	Harmonise item purchase within hospital and clusters	
	Ensure adequate cleaning services and waste management capacity	
		Consider extended or limited re-use of N95 respirators
		Consider alternatives to N95 respirators, e.g. PAPR
		Rationalise the use of N95 respirators (e.g. risk stratify by activity type)
		Obtain alternative sources of mechanical ventilators
		Utilise stockpiled transport ventilators if available
		Mass critical care: use alternative forms of respiratory support (e.g. NIV, HFNC) to replace invasive mechanical ventilation
STANDARDS	Maintain clinical standards and principles of ARDS (e.g. lung protective ventilation, prone ventilation when appropriate)	
	Consider early intubation; avoid NIV in the absence of evidence-based indications	
	Adapt resuscitation and emergency procedural workflows to optimise patient safety and minimise risk of transmission	
	Identify ECMO referral centre, establish referral and transport workflows	
	Establish a hospital outbreak response command centre for effective communication and coordination	
	Inter- and intra-hospital teleconferencing to share experience and knowledge	
	Coordinate hospital ICU efforts with regional and national plans	
	Continue to engage patients’ relatives	
	Utilise public relations and communications resources to build public trust	

*ICU* intensive care unit, *AIIR* airborne infection isolation room, *COVID-19* coronavirus disease 2019, *OT* operating theatre, *PACU* post-anaesthesia care unit, *HCW* healthcare worker, *PPE* personal protective equipment, *PAPR* powered air-purifying respirator, *NIV* non-invasive ventilation, *HFNC* high-flow nasal cannula therapy, *ARDS* acute respiratory distress syndrome, *ECMO* extracorporeal membrane oxygenation

*The planned response should ideally be a phased or tiered response or a continuum-based response which evolves along with the impact of the pandemic

## Key strategies at different phases of a pandemic

When faced with sporadic cases or defined clusters in the community, maximising containment to reduce community impact and buy time for preparations is the key priority. Within healthcare institutions, this is achieved with rapid identification and isolation of suspect or confirmed COVID-19 cases, and strict infection control measures to minimise intra-hospital transmission and prevent incapacitation of essential services. A study from Wuhan, China, reported 41% of COVID-19 cases attributable to hospital-related transmissions, with the majority (70%) being HCWs [3]. In Italy, up to 20% of responding HCWs were also reported to be infected [16], emphasising the need for strict containment measures. If national and regional containment measures fail, healthcare systems are at risk of being rapidly overwhelmed [7]. In the event of sustained widespread community transmission, emphasis then shifts towards supporting essential hospital services, such as critical care and emergency care, to maximise mitigation whilst maintaining containment efforts. The planned response is a continuum and will vary based on the scale and severity of the pandemic [14].

## SPACE: critical care beyond the ICU

### Designating an isolation ICU

**Fig. 1:**
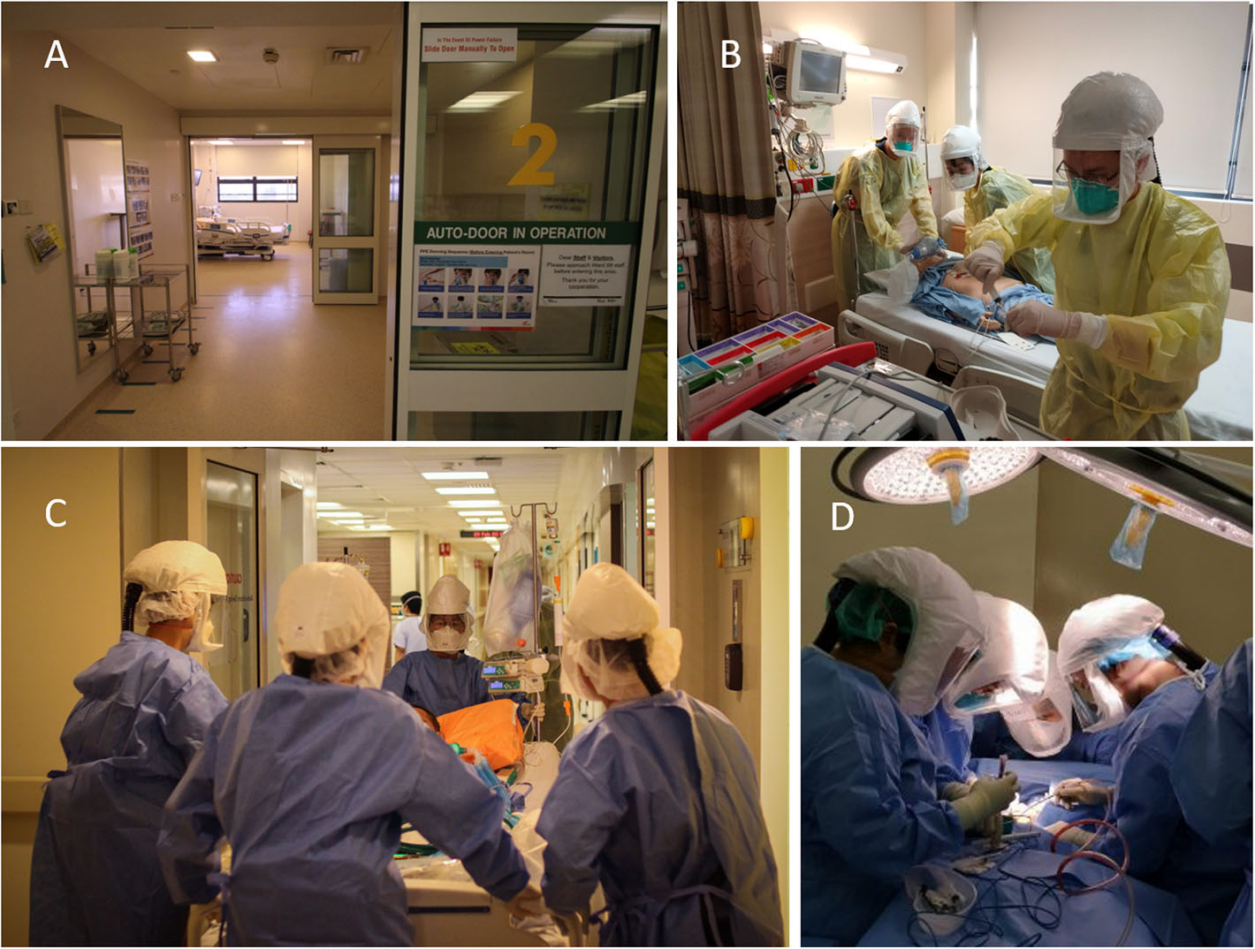
Infection control and prevention. **a** Negative pressure airborne infection isolation rooms (AIIRs) used for our isolation ICUs. **b** Cardiac arrest simulation training with disposable caps, gloves, and fluid-resistant gowns, eye protection along with respiratory protection using a fit-tested National Institute of Occupational Safety and Health (NIOSH) certified disposable N95 filtering facepiece respirator and powered air-purifying respirators (PAPRs). **c** Transport checklists and protocols (movement plans) were determined and rehearsed before actual implementation. **d** Operative procedures performed with PAPR in preparation for the COVID-19 pandemic

Designation of an isolation ICU ward, geographically separated from other clinical areas, allows for concentration and segregation of equipment and staff, contributing to more effective containment. Isolation ICUs should ideally consist of negative pressure airborne infectious isolation rooms (AIIRs). AIIRs are kept at negative pressure relative to surroundings and ventilated with at least 6–12 air changes per hour (Fig. 1a), with any recycled air filtered before recirculation [17]. For hospitals without AIIRs, containment may also be maximised with a dedicated ICU and strict infection control measures. If single rooms are unavailable, cohorted patients should ideally be nursed at least 2 m apart with engineering controls (separation with physical barriers) [18].

### Rapid identification and isolation of suspected or confirmed cases

Rapid identification and isolation of suspected COVID-19 cases requires an efficient triaging protocol and access to rapid diagnostic testing. In our centre, all patients are screened at the emergency department, inpatient wards, and outpatient clinics. Based on screening questionnaires (travel and contact history), symptoms, and preliminary investigations (chest radiographs), patients are stratified into COVID-19 risk levels and admitted into open wards, cohort beds, single room beds, or negative pressure isolation rooms, respectively. With guidance from government authorities, our hospital’s ‘outbreak response command centre’ constantly updates necessary changes to our COVID-19 risk stratification protocols, which are immediately disseminated to HCWs. Diagnostic testing is performed on specimens (pharyngeal swabs, or lower respiratory tract samples if applicable) for SARS-CoV-2 with real-time reverse transcriptase–polymerase chain reaction (RT-PCR)—we emphasise that healthcare systems need to ensure that there is adequate infrastructure and facilities to enable rapid testing.

As with other respiratory viruses, SARS-CoV-2 is believed to be most contagious when patients are symptomatic. However, unlike MERS and SARS [19], transmission of SARS-CoV-2 may occur even with mild or asymptomatic illness [20]. This complicates identification of cases and contact tracing. In Singapore, comprehensive and coordinated contact tracing is done at the hospital and national level to maximise containment efforts [21]. Our suspect case definition is currently based on a combination of travel and contact history, and presence of respiratory illness [22]. Patients who do not fulfil these criteria but have respiratory symptoms or chest imaging suggestive of a respiratory infection are cohorted to designated ‘acute respiratory illness’ wards for surveillance and segregation. Each healthcare system will need to constantly evolve their protocols and suspected case criteria to balance safety, yet optimise resource utilisation [23, 24].

### Maintaining ICU surge capacity

In general, ICUs should be equipped to expand immediately by at least 20% above baseline capacity [14]. During a pandemic, however, significantly higher surge ICU capacity is needed and critically ill patients may need to receive care outside of a traditional ICU [14]. The planned response will depend on available resources, and trigger targets for activation of each phase of response should be defined early.

Alternative sites to care for critically ill patients must be identified early. In our centre, plans are made to allow for the immediate conversion of all isolation AIIRs into ICU beds. Beyond this, patients will be cared for in existing ICU beds. We have also made comprehensive plans for the use of existing monitored beds (e.g. high dependency units, post-anaesthetic care units, operating suites, endoscopy suites) and cohorting of confirmed cases in designated areas. It is important that ICU bed planning should take into consideration the availability of oxygen ports, compressed air supply, clean water, and drainage systems [18]. Concurrent timely discharge to step-down care areas is critical and enables ICU resources to be used effectively. By this time, all elective surgeries and non-essential services need to be suspended so that resources can be quickly redirected.

At the extreme end of the surge continuum, ‘emergency mass critical care practices’ will have to be instituted which may come at a cost of suboptimal standards of care [14, 24]. This may mean that high-flow nasal cannula (HFNC) therapy or non-invasive ventilation (NIV) is used to mitigate acute ventilator shortages, even though they do not constitute preferred management due to infection control concerns (aerosolisation of respiratory droplets). Triage of critically ill patients may become necessary to prioritise ICU resources, and ethical principles must be carefully considered to ensure just and equitable delivery of care for all patients [25–27]. Triaging protocols should not disadvantage patients without COVID-19 who need ICU care. Healthcare systems must find a balance between ‘saving the most lives’ and prioritising care based on the likelihood of clinical benefit [28]. These criteria should ideally be objective, transparent, and publicly disclosed. Authorities should engage the community in this process so that public trust exists when it is most needed [25–27, 29].

## STAFF: maintaining service capabilities and protecting healthcare workers

### Establish infection control measures

Infection prevention and control is essential to protect both patients and HCWs [30]. Experience from SARS demonstrated the vulnerability of HCWs, and an uncontrolled in-hospital outbreak can devastate an entire hospital’s services within days [31, 32]. Critical care HCWs are at high risk considering the higher exposure dose from aerosol-generating procedures and longer periods of patient contact. In addition, the reproductive number for SARS-CoV-2 (between 2 and 2.5) and therefore transmissibility is significantly higher than seasonal influenza [33]. As with other respiratory viruses, it is likely that SARS-CoV-2 spreads predominantly through contact (direct or indirect) and droplet transmission. Though faecal shedding of the virus has been demonstrated, faecal-oral transmission remains unproven [34]. Strict adherence to droplet and contact precautions including hand hygiene, eye protection, and safe donning and doffing of personal protective equipment (PPE) will be the main defences against transmission. A summary of recommended infection control measures is presented in Table 2.

**Table 2:** Summary of infection prevention and control measures

	Infection prevention and control measures
General measures	Develop robust risk stratification criteria Actively identify and isolate patients suspected to have COVID-19 Effective contact tracing Rapid laboratory diagnostic testing Care for suspected or confirmed cases in negative pressure AIIR—patients to wear face masks until transfer to AIIRs Strict hand hygiene and standard precautions Staff PPE requirements **For all patients:** droplet and standard precautions, with additional airborne precautions when performing aerosol-generating procedures **For suspected/known COVID-19 patients**: droplet, contact, and airborne precautions Fit testing for all staff using N95 respirators Staff training (and re-training) for appropriate use, donning, and removal of PPE, with pictorial guides and videos where applicable Stockpile PPE and consumables for infection control Single-use items for patients (e.g. disposable blood pressure cuff) Disinfect shared equipment after use Provision of (disposable) staff scrub suits in isolation wards Appropriate handling of medical waste Hospital issued guidelines for infection prevention, including handling of patient specimens and care of the deceased patient Staff segregation and physical distancing Centrally tracked staff surveillance (e.g. temperature monitoring) and access to designated staff clinics Reduce face-to-face encounters with patients (e.g. video monitoring, telemedicine, wearables for vital sign monitoring) Minimise patient movement or transport Exclude visitors to patients with suspected or known COVID-19 Restrict unnecessary attendance at hospitals (e.g. medical students, members of public, research coordinators) Minimise or postpone elective admissions and operations *Droplet and Contact PPE:* *Surgical mask, eye protection, disposable gown, gloves, and cap* *Droplet, Contact and Airborne PPE:* *N95 respirator (consider PAPR use), eye protection, disposable gown, gloves, and cap*
Aerosol-generating procedures	Perform aerosol-generating procedures only in presence of a clear clinical indication Consider alternative therapy (e.g. inhaled medications by metered dose inhaler and spacer rather than nebulised therapy) Consider conventional oxygen therapy (instead of NIV and HFNC) and early intubation for COVID-19 pneumonia Airborne precautions Issue hospital guidelines on aerosol-generating procedures Consider the use of PAPR if available and staff are trained in its use Procedure to be done in AIIR or single room Limit staff involved in aerosol-generating procedures Limit duration and exposure during aerosol-generating procedures (e.g. stop ventilation before circuit disconnection)
ICU-specific measures	Consider high-efficiency particulate air (HEPA) filters at (Fig. 2) • Expiratory port of breathing circuit • Bag-valve-mask interface • NIV mask interface Use heat and moisture exchanger (HME) instead of a heated humidifier Use closed, in-line suction of tracheal tubes Measures to reduce dispersion of aerosols during intubation (Table 3) Use of single-use equipment (e.g. bronchoscopes) Segregate ICU equipment (e.g. ultrasound machines) Incorporation of infection control measures into ICU workflows (e.g. cardiac arrest and rapid response teams, transport, emergency operations and procedures) In situ simulation sessions

*COVID-19* coronavirus disease 2019, *AIIR* airborne infection isolation room, *PPE* personal protective equipment, *PAPR* powered air-purifying respirator, *NIV* non-invasive ventilation, *HFNC* high-flow nasal cannula therapy, *ICU* intensive care unit

Airborne transmission with respiratory viruses remains controversial [37], although it is likely that any airborne transmission with COVID-19 will be opportunistic in nature [38]. Studies on SARS-CoV and MERS-CoV have highlighted the possibility of airborne transmission with aerosol-generating procedures such as intubation [17], manual ventilation [39], NIV, and tracheotomy [40]. Until more is known about COVID-19 transmission, we recommend airborne precautions during aerosol-generating procedures in all patients (COVID-19 or otherwise) [17]. Disposable caps, gloves and fluid-resistant gowns, eye protection, and respiratory protection using a fit-tested National Institute of Occupational Safety and Health (NIOSH) certified disposable N95 particulate filtering facepiece respirator are used in our centre [17]. Powered air-purifying respirators (PAPRs) are alternatives, but careful doffing and disinfection is necessary to prevent self-contamination [41]. HCWs must doff and dispose of PPE safely within designated areas, with equipment and environmental decontamination enforced.

**Fig. 2:**
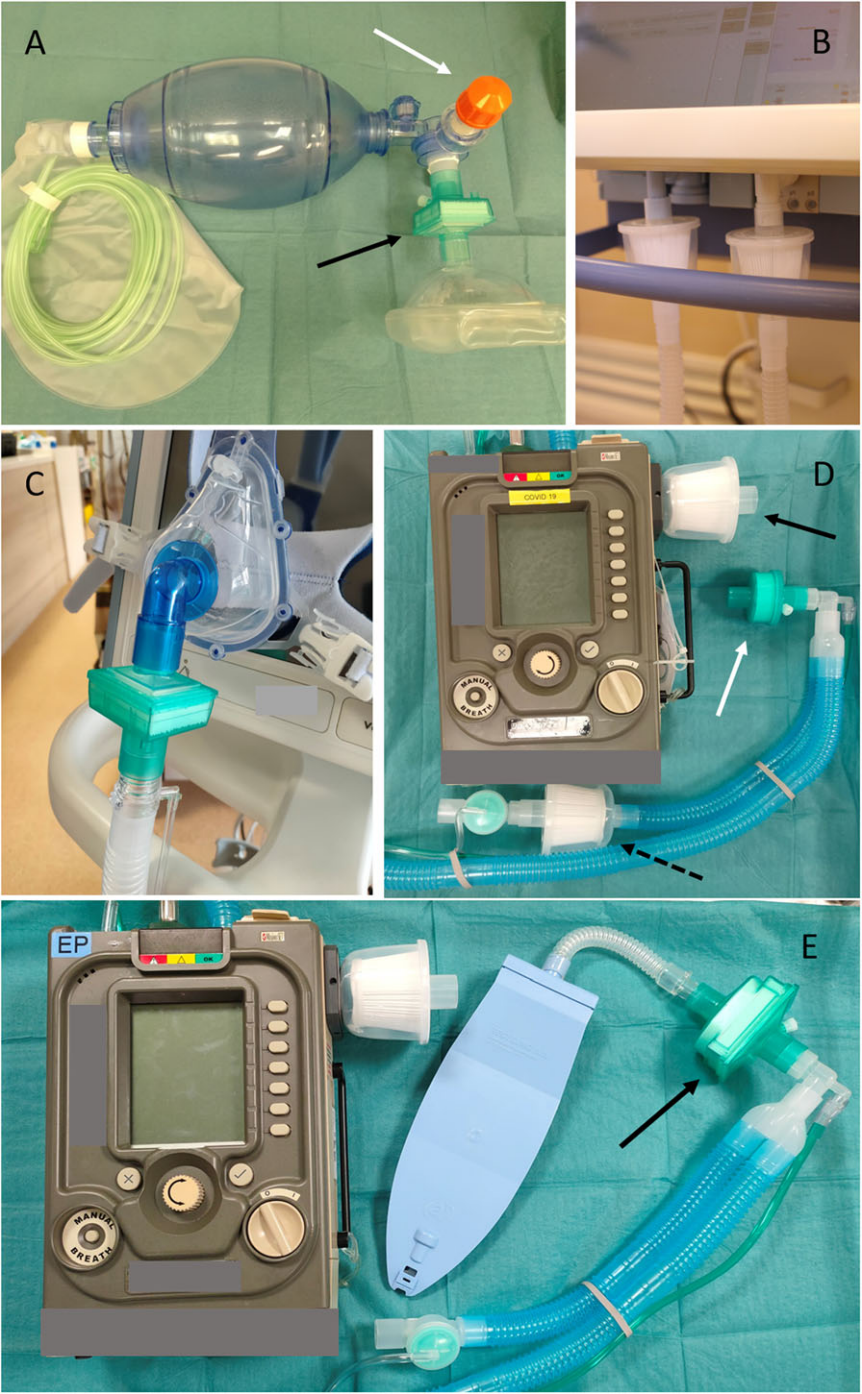
Breathing circuit filters. Connections should be tightly fitted to avoid disconnections during movement. Dead space and circuit resistance will increase with the use of filters. **a** Hydrophobic mechanical filter at the bag-valve-mask interface (black arrow) to reduce dispersion of respiratory droplets during manual ventilation with a positive end expiratory pressure (PEEP) valve (white arrow) to optimise pre-oxygenation. **b** HEPA (high-efficiency particulate air) filters used on the inspiratory and expiratory ports of mechanical ventilators. **c** Hydrophobic mechanical filter used before the external expiratory port on a single-limb non-invasive ventilator circuit. **d** HEPA filters used at the air inlet (solid black arrow) and before the exhalation valve (dashed black arrow) ports of a single-limb transport ventilator circuit. A standard HME (heat and moisture exchanger) (white arrow) is attached at the Y-piece of the breathing circuit. **e** An alternative combination is the use of HEPA filter at the air inlet and a hydrophobic mechanical HME filter (HMEF) (black arrow) at the Y-piece, nearest to the patient

Other potential aerosol-generating procedures (e.g. airway suctioning, nebuliser treatment, bronchoscopy, chest physiotherapy, and HFNC) have not been conclusively established to increase the risk of viral transmission [40]. Nevertheless, we discourage nebulised therapy (metered dose inhalers with or without a spacer are used instead) and use closed in-line suction for tracheal aspiration in our centre. With no clear mortality benefit with HFNC therapy in respiratory failure, our preferred management is conventional oxygen therapy and early intubation [42]. NIV is considered only with evidence-based indications, in view of high failure rates and poor outcomes seen with MERS-CoV [43]. However, we do recognise that in the event of severe ventilator shortages, these modes of respiratory support may have to be utilised—ideally with strict infection control measures. In our institution, mechanical high-efficiency particulate air (HEPA) or pleated hydrophobic filters are fitted to mechanical ventilators at expiratory ports, as well as NIV mask and bag-valve-mask interfaces (Fig. 2). It should be noted that airway resistance and circuit dead space may be increased as a result [44–47].

### Education and re-training

Education is important for effective infection control, upskilling HCWs, and identifying logistical and technical challenges with routine or ICU care. HCWs should be taught to inspect, disinfect, and dispose of PPE safely, and periodic refresher re-training is required to ensure staff readiness and proficiency. In our centre, an online critical care refresher course was organised for non-intensivist physicians, with voice-annotated lectures on ICU management and instructional videos on ventilator set-up and troubleshooting.

From our experience, simulation training has been invaluable in ensuring the preparedness of HCWs [48, 49]. We instituted ‘just-in-time’ inter-professional and in situ simulations. These included resuscitation and rapid response team training, extracorporeal membrane oxygenation (ECMO), transport of critically ill patients, and procedures like tracheostomy (Fig. 1), performed with full airborne precautions and PAPR. These helped to recognise and address unexpected problems, which led to downstream rectification. For example, cardiac arrest simulations revealed several violations of infection control during cardiopulmonary resuscitation, and challenges such as difficulties in auscultating for breath sounds while using PAPR. This triggered the recommendation for infection control re-training and posters for PPE doffing to be placed in all isolation wards. End tidal carbon dioxide monitoring was also used to confirm endotracheal tube placement for all intubations. Transport with full-feature ICU ventilators also revealed problems with limited internal battery lifespan, and physical bulk even with the largest available cargo lifts. Protocol changes to use transport ventilators fitted with HEPA filters were then made. Multidisciplinary peer-review processes were also held after each procedure with COVID-19 patients, where HCWs and relevant stakeholders were debriefed and process improvements discussed.

### Segregation and surveillance

HCWs should be segregated to reduce the risk of cross-transmission. This allows for service continuity, should a team be quarantined. In our centre, HCWs caring for COVID-19 patients are also not permitted to care for other patients and are relieved of outpatient responsibilities. In our ICUs, we allocated ‘frontline’ teams of doctors, nurses, and allied health staff, with defined geographical coverage for each team. Teleconferences replaced face-to-face meetings. HCWs were instructed to record temperature surveillance readings twice a day and submit these to an online platform that is centrally tracked to facilitate early detection of any intra-hospital outbreak. HCWs with fever or respiratory symptoms are also required to report to designated staff clinics, with COVID-19 testing and/or stay home instructions implemented as required.

### Maintaining manpower capability

With the need for strict infection control measures, routine procedures in the ICU will require additional manpower and time [49]. In our isolation ICU, the nurse-to-patient ratio was increased to approximately 1.5:1, with critical care nurses focused on providing care and supported by non-critical care nurses who assisted in preparing drugs and equipment outside the AIIRs. Infection control specialty nurses assisted ICU staff in appropriate donning and doffing of PPE. In addition, doctors and nurses with previous critical care experience were also reassigned from other departments to extend frontline coverage to 24 hours a day.

In the event of widespread community transmission, critical care manpower capabilities will be easily overwhelmed. It will then be necessary to draw manpower from various departments and divisions in the hospital. In our centre, critical care crash courses are being held continuously for non-critical care nurses with online learning materials, instructional videos, and hands-on practicums supervised by critical care nurses in non-isolation ICUs. Manpower is also being drawn from nurses with critical care experience, recently retired ICU nurses, and private hospital nurses. Nurses from various subspecialty centres within the extended hospital campus can augment non-critical care nursing roles, such as infection control [50]. Critical care intensivist- or nurse-to-patient ratios should be re-evaluated frequently, guided by clinical demands, provider experience, and available support by ancillary staff [50]. With critical shortages in manpower, intensivists and ICU nurses may have to take up a supervisory role, with non-critical care HCWs providing direct care to patients.

High workload and anxiety over disease transmission can result in significant physical and mental fatigue [51]. To mitigate this, we established open communication channels and rapid dissemination of information to keep staff informed of new developments. Psychological support resources and a helpline are also made available. Sections of a community hospital building were repurposed as optional temporary staff quarters. Despite best intentions, ‘presenteeism’, the act of coming to work whilst ill, potentially increases the risk of intra-hospital transmission [52]. Policies must ensure that HCWs seek early medical attention and stay home when ill.

## SUPPLIES: equipping ICUs

In a disease outbreak, requirements for equipment and supplies including PPE increase tremendously. Allied health and support services, including pharmacy, laboratory, and respiratory therapy services, must be engaged in advance to identify the highest priority supplies and medications required. To avoid supply chain vulnerabilities, alternative agents and supply channels should be identified [53]. Equipment, supplies, and pharmaceutical stockpiles should be harmonised as far as possible within the hospital and between regional hospitals [50]. Single-use items (e.g. disposable laryngoscope blades and bronchoscopes) may be preferable. For reusable items, it is important to ensure adequate capacity for prompt disinfection and sterilisation.

Careful use of PPEs can help to conserve supplies required for infection control. Extended use and limited re-use of N95 respirators may be implemented with appropriate precautions (e.g. strict hand hygiene, reusable face shield over an N95 respirator) [54]. Other options include alternative respirators (e.g. elastomeric respirators, PAPRs) or prioritising use of respirators by activity type (e.g. aerosol-generating procedures) while masking symptomatic patients [55].

Our centre has additional full-feature invasive mechanical ventilators and transport ventilators ready for deployment to allow immediate expansion of ICU services. If greater capacity is required, ventilators from operating theatres and other monitored units and transport ventilators from national stockpiles are available. Apart from an expected surge in demand for drugs such as sedatives, analgesic agents, and neuromuscular blockade agents, specific drugs will also be required during a pandemic. The route of drug delivery must be considered. For example, lopinavir/ritonavir, usually given as a tablet, may need to be administered in a syrup formulation for critically ill patients. Crushing the tablet form may result in poorer efficacy due to interactions between the two active components of the drug [56].

Finally, significant amounts of biohazard waste will be generated while caring for critically ill patients. Adequate waste management capacity is necessary to upkeep a safe environment for HCWs and patients [57]. Environmental cleaning appears to be effective against SARS-CoV-2 [58]; however, terminal cleaning will need to keep pace with clinical services to maximise bed capacity.

## STANDARDS: ensuring quality clinical care

### Management of critical illness from COVID-19

We still have a poor understanding of clinical features of critical illness in COVID-19. Based on the current evidence, the spectrum of disease severity appears to vary significantly from asymptomatic or mild symptoms to critical illness with a high mortality rate [4]. Critical illness appears to develop most commonly between 1 and 2 weeks after symptom onset [3, 5]. Ground glass opacities are typical findings on chest imaging, with basal consolidation seen in some patients [59, 60]. However, chest radiograph or computed tomography (CT) imaging can be normal, even in patients who subsequently develop critical illness [61]. In addition, patients can develop rapid deterioration and progression to ARDS [62]. Risk factors for critical illness include advanced age (≥ 60 years) and pre-existing comorbidities (cardiovascular disease, cerebrovascular disease, chronic pulmonary disease) [3, 61, 63]. Biochemical ‘markers’ including significant lymphopenia, elevated lactate dehydrogenase or d-dimer, and neutrophilia are associated with more severe disease [3, 5, 63]. Nosocomial or secondary infections appear to be common (14–31%) in critically ill patients [4, 5]. It remains unclear if COVID-19 can result in fulminant myocarditis [63].

Until more evidence emerges, management of COVID-19-induced ARDS should not differ from conventional ARDS management [64, 65]. These include lung protective ventilation, a fluid conservative strategy when appropriate, and early initiation of prone ventilation for severe ARDS [66–68]. When appropriate, early neuromuscular blockade may be considered for moderate-severe ARDS [69]. Referral to an ECMO centre, for refractory hypoxemia or if lung protective ventilation cannot be applied, remains an option. Corticosteroids should not be routinely administered [70], with concerns of delayed viral clearance or worse patient outcomes extrapolated from studies with SARS and MERS-CoV [71, 72]. Whilst there is no proven anti-viral therapy for COVID-19 to date, ongoing trials include the use of remdesivir, which has shown some promise in in vitro studies [73]. More research is urgently needed to fill knowledge gaps in terms of disease clinical course, prognostic markers, treatment options, and transmission dynamics, which will be critical for ICU management and resource planning.

#### Intubation and airway codes

The principles of airway management are, firstly, to optimise patient safety and, secondly, to minimise the risk of transmission to HCWs. Table 3 summarises the recommendations for intubation of the COVID-19 patient. Physicians should have a lower threshold to call for help in a predicted difficult intubation. This allows the airway code team additional preparation time (donning of PPE) and minimises multiple attempts at intubation and manual ventilation. In our centre, dedicated airway kits were placed in isolation wards and in the emergency department for use on suspect and confirmed COVID-19 cases. Additional items such as N95 respirator masks, goggles (eye protection), and endotracheal tube (ETT) clamps, to limit aerosolisation following intubation, were added to the difficult airway equipment.

**Table 3:** Recommendations for intubation and transport of a suspected/known COVID-19 patient

	Optimise patient safety	Infection prevention and control
Preparation	Early identification of patients requiring intubation [35] Formulate airway plans A, B, C, D Don PPE with airborne precautions Prepare all equipment for intubation • Airway • Breathing devices, e.g. bag-valve-mask device • Breathing circuit	*Intubate within an AIIR [17, 36] PPE and airborne precautions for all staff [17, 36] HEPA filter to reduce circuit and environmental contamination (Fig. 2a)
Intubation	Preoxygenation for 5 min, with ‘head-up’ positioning when possible Consider PEEP valve with bag-valve-mask pre-oxygenation Consider nasal cannula (15 L/min) for apnoeic oxygenation Intubation by the most experienced operator Use video laryngoscope to optimise view through PAPR or goggles	Ensure good mask seal Avoid HFNC for pre-oxygenation Rapid sequence induction—minimise need for face mask ventilation [35] Small tidal volumes if ventilation unavoidable [35] Ensure full paralysis to reduce coughing [35]
Post-intubation	Confirm tracheal tube position with capnography (difficult auscultation with hooded PAPR)	Positive pressure ventilation to be initiated only after cuff is inflated Sedation and paralysis to reduce coughing
Transport of the intubated patient	Consider if transport is necessary Sedation and paralysis to reduce risk of coughing or inadvertent self extubation	HEPA filters for circuit and transport ventilator (Fig. 2) Place ventilators on standby mode and clamp tracheal tube for the period of disconnection [36] Adhere to a designated route with minimal contamination and exposure to other clinical areas

*COVID-19* coronavirus disease 2019, *PPE* personal protective equipment, *PEEP* positive end expiratory pressure, *PAPR* powered air-purifying respirator, *AIIR* airborne infection isolation room, *HFNC* high-flow nasal cannula, *HEPA* high-efficiency particulate air

*Intubation should ideally be performed in an AIIR for suspected or known COVID-19 patients

#### Intensive care outreach

All cardiac arrests in the hospital are managed with full PPE in the first instance [74]. A designated cardiac arrest team was assigned for the isolation wards in our hospital, segregating them from other geographically based teams. The remaining cardiac arrest teams were re-structured to exclude contribution from the emergency medicine department, allowing for further segregation of manpower for emergency medicine services. Rapid response and outreach services should also be streamlined to reduce staff movement throughout the hospital. Should ICU resources be overwhelmed, these services may have to cease or be re-delegated with supervision from critical care teams.

#### Transport of critically ill patients

Table 3 lists recommendations for transport of intubated COVID-19 patients. A transport ventilator is used for our transports (Fig. 2). In the event of ventilator shortages, a full-feature ICU ventilator may be used instead; advantages include reduced circuit disconnections and the ability to deliver advanced modes of ventilation. However, additional oxygen tanks will have to be prepared as ICU ventilators are not able to utilise atmospheric air. It is important to ensure that battery life (external and/or internal batteries) is sufficient to last the entire duration of transport, procedure, or imaging.

#### Emergency procedural workflows

Workflows and service continuity for isolated patients who require emergency operations or interventional procedures should be determined. Dedicated operating theatres, radiology, and interventional suites, along with respective subspecialty teams, were designated in our institution [75, 76].

### Effective communication and coordination

Effective communication is essential in a pandemic response. Information should be disseminated to all HCWs in a timely fashion, and two-way communication should be established. In our centre, information and protocol updates are disseminated to all HCWs daily by email and hospital intranet. In addition, ICUs will need to work closely with nursing services, infection control, supplies/engineering/cleaning services and other departments to harmonise efforts for a coordinated response. In our institution, an ‘outbreak response command centre’ involving key appointment holders discusses anticipated challenges and reviews protocols regularly. In Singapore, several ICU-led inter-hospital video conferences have also been organised to share knowledge. Finally, good coordination between local or regional hospitals, guided by government or national organisations, is a key component of the pandemic response with effective resource allocation [77].

### Family engagement

Family engagement is important as visitors are largely excluded from isolation wards [78]. Effective communication is necessary to address concerns and manage expectations, or to help determine goals of care in the critically ill. Public relations departments and social workers should be involved to facilitate communication with family members or the public media, to minimise misunderstanding and build trust and confidence in the healthcare system [79].

## Conclusion

The COVID-19 pandemic will place extraordinary and sustained demands on critical care services. Critical care providers must take the lead to initiate planning and rapidly implement measures to ensure that healthcare services are maintained. It is equally important to maintain critical care services for non-COVID-19 patients, protect HCWs, and consider the ethical and social implications of triaging during a crisis.

## Data Availability

Not applicable

## Abbreviations

COVID-19: Coronavirus disease 2019
SARS-CoV-2: Severe acute respiratory syndrome coronavirus 2
ARDS: Acute respiratory distress syndrome
MERS-CoV: Middle East respiratory syndrome
SARS: Severe acute respiratory syndrome
ICU: Intensive care unit
HCW: Healthcare worker
AIIR: Airborne infectious isolation room
RT-PCR: Real-time reverse transcriptase–polymerase chain reaction
HFNC: High-flow nasal cannula therapy
NIV: Non-invasive ventilation
PPE: Personal protective equipment
NIOSH: National Institute of Occupational Safety and Health
PAPR: Powered air-purifying respirator
HEPA: High-efficiency particulate air
ECMO: Extracorporeal membrane oxygenation
CT: Computed tomography
